# Supplementary material for: A stochastic analysis of the interplay between antibiotic dose, mode of action, and bacterial competition in the evolution of antibiotic resistance
Source: PLoS Comput Biol. 2023 Aug 14;19(8):e1011364. doi: 10.1371/journal.pcbi.1011364 (PMC10449190; doi:10.1371/journal.pcbi.1011364)
Supplement: Attachment — Submitted filename: Review_Plos_ComBio.pdf [file pcbi.1011364.s002.pdf]

Summary of the work:

In this work, the authors develop a stochastic model of a population subjected to drug treatment. This model aims to investigate the evolution of antibiotic resistance within a host. To this end, the authors focus on three quantities:

- The resistant survival probability
- The resistant sub-population size at the end of a treatment
- The carriage time after treatment

These quantities are explored by a combination of numerical and analytical tools.
One of the original features of this work is the distinction between biostatic antibiotics, which impede bacterial division, and biocidal antibiotics, which kill bacteria. Another originality is that the authors distinguish two types of density regulation, either on the birth rate or on the death rate. These distinctions allowed the authors to investigate the dynamics of resistance evolution under different scenarios (e.g., biostatic antibiotic and density regulation on the death rate) and assess the impact of different antibiotic modes of action and density regulation on the evolution of resistance.

General comment:

I find this work very interesting, and I enjoyed reading it. First, given the threat to public health posed by the evolution of antibiotic resistance, this theoretical work is timely. Second, given that the development of new antibiotics is struggling, I find evaluating the impact of different drug modes of action (i.e., biostatic versus biocidal) is relevant. This aspect has been neglected, although it represents a possible angle of attack to fight antibiotic resistance. I do not have any serious concerns about this work, although I think the discussion could be strengthened (I elaborate below). I add some minor points and suggestions that the authors can address if they find them relevant.

Major points:

A) In my opinion, the discussion could be strengthened. If I am correct, the authors do not place their results in a clinical context. Whereas the authors claim to investigate the within-host dynamics of antibiotic resistance, they only mention *in vitro* experiments (lines 425-449). I think it would be great to cite some meta-analyses in which the efficiency of biostatic and biocidal drugs in eradicating a given infection is compared (e.g., Johannes Nemeth, Gabriela Oesch, Stefan P. Kuster, Bacteriostatic versus bactericidal antibiotics for patients with serious bacterial infections: systematic review and meta-analysis, *Journal of Antimicrobial Chemotherapy*, Volume 70, Issue 2, February 2015, Pages 382–395, https://doi.org/10.1093/jac/dku379). These meta-analyses, which focus on the ability to eradicate infection and not on the evolution of resistance, do not find significant differences between biostatic and biocidal antibiotics (except in particular cases). Your work shows that there is a difference in the evolution of resistance, and it would be interesting to highlight it. In short, I think many readers will ask: is it better to use biocidal or biostatic antibiotics to prevent the evolution of resistance?

B) The authors compare two drug modes of action, namely biostatic and biocidal. This comparison is not novel since it has already been investigated in reference [39]. It would be interesting to compare the results of this work with those of reference [39]. Are they complementary? Do they contradict each other? Do they favor biostatic or biocidal antibiotics to prevent antibiotic resistance? Etc. I would also like to point the authors to the following reference, which is also relevant for a potential comparison: The risk of drug resistance during long-acting antimicrobial therapy, A Nande, AL Hill, *Proceedings of the Royal Society B*, 2022.

C) From line 395 to line 406, the authors discuss their parametrization choice. However, the authors should mention whether the values are coherent with experimental observations. For example, is a resistance cost equal to ~10% relevant? The auhors could cite the following meta-analysis: Melnyk AH, Wong A, Kassen R. The fitness costs of antibiotic resistance mutations. *Evol Appl.* 2015. Same comment for the MIC differences between resistant and sensitive bacteria. Also, I think the authors should discuss whether the resistance cost depends on the drug mode of action, as some readers may wonder if comparing biostatic and biocidal antibiotics for the same parameter values is relevant. Suppose I carry out two experiments to eradicate one bacterial population: one with a biostatic antibiotic and another with a biocidal antibiotic. The evolutionary pathways to resistance will likely be different in the two cases. Here is an interesting reference: Chevereau G, Dravecká M, Batur T, Guvenek A, Ayhan DH, et al. (2015) Quantifying the Determinants of Evolutionary Dynamics Leading to Drug Resistance. *PLOS Biology* 13(11): e1002299.

Minor points:

1) The authors present their model as a bacterial population within a host. I wonder whether it is necessary to specify "within a host." My concern is that it is unclear what distinguishes the authors' model from a model of an *in vitro* bacterial population. Also, as specified from line 419 to line 424, the authors' model does not include pharmacokinetics, an essential ingredient of within-host bacterial populations subjected to antibiotics.

2) Instead of specifying the quantities the authors focus on in the title, why do they not mention the comparisons they made, namely different drug modes of action and types of density regulation? This would make the title more attractive.

3) lines 32-33: I suggest the authors go through the following paper: The risk of drug resistance during long-acting antimicrobial therapy, A Nande, AL Hill, *Proceedings of the Royal Society B*, 2022.

4) lines 63-64: Maybe I am wrong, but this sentence sounds like the resistant sub-population goes extinct with a probability equal to one in the drug-free phase. The extinction probability is not equal to one, right? If the resistant sub-population size is substantially larger than the sensitive sub-population size at the end of treatment, the latter would probably die out even if the sensitive individuals have a selective advantage, right? Could the authors provide the reader with an idea of the extinction probability?

5) line 91: "the overall birth and death rate" = the *per capita* birth and death rate?

6) line 116: I would replace "maximal growth rate" with "net growth rate." In microbiology, the maximal growth rate usually refers to the maximum of the curve (dN/dt)/N, which is, in the case of a lag-free logistic growth, obtained at time t=0 and not "measured during the exponential phase" (line 116).

7) Figure 2: The authors could add "grow" above 0 and "decline" below 0 to help the reader identify the antibiotic concentration regimes leading the bacterial population to extinction.

8) line 136: The word "cell" is lost all by itself in a line.

9) lines 137-146; Stochastic simulations: More information could be brought into this section. For example, what algorithm did the authors make use of? Is it a Gillespie algorithm, which is exact, or did the authors use an approximate method such as tau-leaping? Also, it would be useful to provide a pseudo-code where you specify every step of your algorithm (i.e., initialization, iteration, etc.) for

readers unfamiliar with C++. The advantage of writing a pseudo-code is to allow every reader to write their code with their favorite programming language. The pseudo-code does not need to be in the main text but could be in the supporting information.

10) lines 162-163: I was very surprised by this sentence. How can standing genetic variation and *de novo* mutations yield similar results? This sentence may sound weird to any reader with the following reasoning: a perfect biostatic antibiotic fully prevents sensitive microbes from dividing so that the sensitive birth rate equals 0. In such a case, it is clear that no *de novo* mutants appear. A biocidal drug does not prevent sensitive microbes from dividing. Although it drives the sensitive bacterial population to extinction faster than a perfect biostatic drug (at least for large enough drug concentrations), a large mutation rate could induce some appearances of mutants, which would find themselves in a situation in which they are favored by natural selection since they are less impacted by the drug than the sensitive microbes. Could the authors comment on that?

11) lines 165-167: The authors could add that a deterministic approach does not allow extinction modeling.

12) Figure 3: Could the authors add to the caption over how many stochastic realizations the data points are averaged? I know the authors wrote in line 143 that the number of stochastic realizations equals 10^6, but including it in a caption is important. Also, could the authors confirm that $\gamma = \gamma_S = \gamma_R$ and XR(0)=1? Are there too many parameters to be added to the caption instead of “Parameter values are the same as in Fig. 2”? Same comment for the other figures.

13) lines 365-366: Could the authors remind the reader of how they scale? Also, the word “intrinsic growth rate” is unclear to me. Is it the difference between the intrinsic birth and death rates?

14) lines 385-388: This sentence is quite long, and I am not sure I understood it. It is not clear to me how it supports the previous sentence. There is a similar reasoning in lines 186-188. How can two populations have the same deterministic dynamics if one has large birth and death rates and the other has low birth and death rates?

15) lines 390-392: This sentence made me realize that the authors do not provide insight into the mean extinction time of a sensitive bacterial sub-population subjected to antibiotics. Since the authors claim that biocidal antibiotics drive sensitive sub-populations to extinction faster than biostatic antibiotics, it would be interesting to provide some timescales. For example, the mean extinction time under a perfect biostatic antibiotic is given approximately by log(K)/δS. How does the mean extinction time under biocidal antibiotics compare to log(K)/δS? Do biocidal antibiotics drive the sensitive sub-population to extinction faster than biostatic antibiotics at all drug concentrations?
