# Supplementary material for: A stochastic analysis of the interplay between antibiotic dose, mode of action, and bacterial competition in the evolution of antibiotic resistance
Source: PLoS Comput Biol. 2023 Aug 14;19(8):e1011364. doi: 10.1371/journal.pcbi.1011364 (PMC10449190; doi:10.1371/journal.pcbi.1011364)
Supplement: Attachment — Submitted filename: responsePDF.pdf [file pcbi.1011364.s003.pdf]

Stochastic antibiotic resistance dynamics under biostatic and biocidal treatments: Resistant survival probability, size at the end of treatment, and carriage time after treatment

Peter Czuppon, Troy Day, Florence Débarre, François Blanquart

(PLOS Computational Biology)

[1] Dear Dr. Czuppon, {ed_general}

Thank you very much for submitting your manuscript "Stochastic within-host dynamics of antibiotic resistance: Resistant survival probability, size at the end of treatment and carriage time after treatment" for consideration at PLOS Computational Biology.

As with all papers reviewed by the journal, your manuscript was reviewed by members of the editorial board and by several independent reviewers. In light of the reviews (below this email), we would like to invite the resubmission of a significantly-revised version that takes into account the reviewers' comments.

In light of the reviewer comments and from our own reading of the manuscript, it appears that the manuscript as written fails to meet the "biological signficance" threshold required by the journal. It is not clear whether the numberical and anlytical results are applicable to the real-lfe situation,

We would like to thank you and the referees for the thoughtful comments. We have considerably revised our manuscript to both emphasise the purpose of our work (provide analytical and simulation results illuminating the stochastic phase of antibiotic resistance emergence), and explain in detail how the chosen parameters reflect biologically relevant scenarios. We believe that these additions demonstrate more clearly how our results are applicable to realistic biological scenarios. The emergence of antibiotic resistance is in many cases a stochastic process, as a single cell transmitted to the host, or mutated to resistance, is at the origin of the resistant subpopulation. This makes our work a relevant addition to traditional 'pharmacokinetics-pharmacodynamics' models, which do not consider stochastic effects. The importance of stochasticity for the emergence of antibiotic resistance was recently acknowledged and empirically demonstrated (Alexander and MacLean, 2020).

The default parameter set used in the main text is motivated by a bacterial systemic infection (Grant et al., 2008) and empirical estimates of antimicrobial response curves (Regoes et al., 2004). In addition, we now also evaluated a second parameter set, which corresponds to *Escherichia coli* dynamics in the human gut (Poulsen et al., 1995), in the Supplementary Material, Section G. This second parameter set describes by-stander selection and is highly relevant because of its importance as a source for resistance evolution (Tedijanto et al., 2018). The results obtained from these two parameter sets are qualitatively very similar.

Lastly, we emphasise once more that our model is biologically relevant in the sense that it explores bacterial dynamics in response to antibiotics from a generalized perspective. We define a large number of possible mechanisms that may affect the evolution of resistance, e.g. the specific form of density dependence, the antibiotic mode of action, and the competition between bacterial cells. Such a general model has not been studied at this level of detail before.

[2] whether the parameters chosen are realistic {ed_1}

We have added a new section to justify the parameter choices (starting in line 159). Yet again, we would like to emphasize that the strength of our manuscript is actually that the results are independent of a specific choice of parameters.

[3] and how the the novel theoretical development are called by failures of simpler models. {ed_2}

Our work is guided by the absence of stochastic results on antibiotic resistance dynamics; previous models do not fail, but simply were not meant to address the question of probability of emergence of antibiotic resistance, except for some recent work that we state in the main text (e.g. Day and Read (2016); Marrec and Bitbol (2020)). The research question defines the methodology. If one aims at estimating the efficacy of different antibiotic treatments at killing bacteria, potentially including processes involving large pre-existing resistant subpopulations, then deterministic pharmacokinetic/-dynamic models are appropriate. If one is interested in the dynamics that lead to the emergence of antibiotic resistance from a single cell (or a small resistant subpopulation), which is the focus of our work and a common scenario, then a detailed stochastic description of the underlying processes is necessary. As detailed in our response to comment [6] from referee 1, deterministic models are by design not able to address this question. In deterministic models resistance either emerges if the resistant mutant has a higher growth rate than the sensitive strain, or it does not. This observation corresponds to the concept of the mutant selection window. Our stochastic analysis provides a more detailed picture of this parameter regime and in particular generates estimates for the antibiotic concentrations that yield the highest risk of resistance emergence.

[4] Therefore, a significant revision would be required for the manuscript to be appropriate for the journal. {ed_3}

Once again, we thank you and the reviewers for the thoughtful comments and helpful suggestions that improved the accessibility, readability and biological relevance of our manuscript. In brief, the most important changes are:

- extended biological justification of the default parameter sets (new section "Parameterization");
- stronger focus on our main result, the resistant survival probability, and a better distinction to deterministic models;
- discussion of related theoretical and clinical findings about biostatic and biocidal treatments;
- analysis of a second parameter set in the Supplementary Information;
- change of title to emphasize more the study of biostatic and biocidal drugs.

Reviewer 1

[5] This work performed full stochastic modeling of how resistant and sensitive populations grow and die during antibiotic treatment. Authors carefully considered various processes, e.g., density regulation, modes of action, transmission, immune response, etc. The resulting mathematical model is extensive, described in detail in Sup Mat (in about 35 pages!). I think this model could be useful to the community. {R1_general}

We thank the referee for this overall positive assessment of our work.

[6] However, as it is written now, it is very hard to understand the focus of the work. After reading a whole article, it is still not clear why this approach is better than previous work. Stochastic modeling could be more realistic. There are various processes (as considered here) that indeed take place during antibiotic treatment. While conventional pharmacodynamic models are inherently deterministic and could be potentially limited, they extensively use experimental data, are practical and are widely used. It is not clear why the authors' framework is better. For example, under what conditions stochastic modeling is relevant? I think that authors should clarify and emphasize the new insight that this approach was uniquely position to provide, which previous work failed to do. {R1:2}

We thank the referee for this comment. We might not have explained clearly enough the context and objectives of our study. Our model is not aimed at using specific experimental data to explain dynamics and estimate the efficacy of a particular antibiotic treatment, which is where deterministic pharmacodynamic and -kinetic models are commonly used (to the best of our knowledge). Rather, the focus of our work is the derivation and biological interpretation of the survival probability of a small resistant subpopulation during treatment, irrespective of the specific pathogen and antibiotic. A deterministic model cannot serve this purpose: in deterministic models, resistant cells will either grow or decline, but the question of how stochastic birth and death affect their fate is out of the scope of a deterministic model. However, stochasticity is an important factor when the number of resistant cells is small, as empirically shown (Alexander and MacLean, 2020). Previous work that focused on the question of resistance survival, e.g. Ankomah and Levin (2014); Day and Read (2016); Scire et al. (2019); Marrec and Bitbol (2020), use stochastic models to compute numerically the survival probability of a resistant subpopulation. Our originality is the analytical solution of this quantity.

In this context, our model is the first one to analytically derive these probabilities, which allows us to identify the concentration that maximizes the risk of resistance evolution without extensive numerical simulations and fixed parameter choices. This is important knowledge if one wants to inform treatment strategies based on theoretical considerations.

In addition to the survival probability, we also investigate the size of the resistant subpopulation at the end of treatment and the carriage time of resistance after treatment has ended. These are important quantities if one aims to incorporate the within-host dynamics into epidemiological

models of antibiotic resistance at the between-host scale, which to the best of our knowledge has not been done yet.

In response to this comment by the reviewer (and to comments [18] and [19] by Reviewer 3), we have changed the title of our manuscript to "A stochastic analysis of the interplay between antibiotic dose, mode of action, and bacterial competition in the evolution of antibiotic resistance" to avoid suggesting that the purpose of our model is to replace deterministic models in the context of inference from actual patient data. Additionally, we have clarified the goal of our study more precisely in the Introduction, in particular the difference with deterministic PK/PD models (lines 32ff.).

[7] There are some numerical data shown for some parameter ranges. But, without discussing why those parameters were chosen and relevant, it is hard to evaluate the importance of the findings. In fact, there are a lot of basic parameters that were taken as it is and were not tuned (e.g. Table A2). It seems that there were some biological assumptions when choosing those values (e.g., why is the birth rate of resistant strain is lower than that of sensitive strain but death rate the same?). Authors should clarify these issues up front. {R1_general3}

We have added a new section about the parameterization of our model (starting at line 159). The parameter set of the main text is chosen in accordance with demographic parameters from bacterial systemic infection (Grant et al., 2008). In addition, we now also simulate all the results for parameters plausible for commensal *Escherichia coli* in the human gut (Section G in the Supplementary Information). The qualitative results are very similar between the two data sets. However, we would like to stress that the purpose of our model was not to model a specific pathogen-drug combination, but to obtain general, parameter-independent predictions about bacterial dynamics under antibiotic treatment, especially the resistant survival probability.

We modeled the fitness cost as a reduction in birth (division) rate and not as an increase in death rate, because any substantial death rate represents outflow from the body compartment (e.g. gut), which should affect both sensitive and resistant strains equally, or the action of the immune system which may also not be preferentially targeted to the resistant strains. In contrast, the fitness cost on the division rate is largely documented *in vitro* from competitive growth assays as reviewed in Melnyk et al. (2015). We emphasize though, that our formulas are derived in the more general case and therefore also cover the case of fitness effects mediated through different death rates.

Reviewer 2

[8] The authors study the stochastic dynamics of a pathogen population comprising a sensitive and a resistant strain that is subjected to antibiotic treatment. The focus is on the probability of survival of the resistant strain until the end of the treatment, as well as on the time required for the sensitive strain to subsequently replace the resistant strain. The main text describes selected results for a few typical scenarios. An extensive supplement contains mathematical derivations and explores the robustness of the main results with regard to modifications of the modeling assumptions. Overall, this is a thorough and well-written study of an important problem that is principally suited for publication in PLoS Computational Biology. {R2_general}

We thank the reviewer for their positive comments and assessment of our work.

Nevertheless, I believe that a revision addressing the points listed below would considerably increase the impact and usefulness of the work.

[9] 1. To what extent can the four scenarios in Table 1 be mapped to actual pathogen-drug combinations? While I assume that the distinction between biostatic and biocidal drugs can be made on the basis of the mechanism of drug action, is it known under what conditions the competition between bacteria affects primarily the birth or the death of cells? {R2:1}

The four scenarios are difficult to map to actual pathogen-drug combinations for the reason that the referee suspects; it is not entirely clear if bacterial competition affects the birth or the death rate. This question, whether density regulation affects the growth or the death rate, has largely been overlooked, perhaps because in deterministic models the distinction does not matter. In fact, when designing our stochastic model we looked for empirical evidence for the predominance of one or the other form of density regulation within a host, e.g. the gut environment.

The model of birth competition where bacterial density reduces the birth rate is intuitively explained by resource competition, one of the oldest observation of bacterial density regulation (Monod, 1949). The model of death competition where bacterial density increases the death rate can be mapped to the production of antimicrobials by some bacteria, which kills neighboring bacteria (Brown et al., 2009; Hibbing et al., 2010; Granato et al., 2019; Niehus et al., 2021). The produced antimicrobials can either be highly specific, only affecting a specific strain, or be very general, killing a large number of bacterial species. These are just two examples of the plethora of competition mechanisms between bacteria (Hibbing et al., 2010). In a host, for example in the human gut, many of these processes interact. Growth or death can be affected by a complex mix of temporal fluctuations in nutrient availability, spatial structure, and different resources. It is therefore unclear (at least to us) which of the competition mechanisms dominate.

In response to this comment, we have now added these two examples of bacterial competition, starting in line 89. We also mention that the relative importance of these two processes within a host is not yet understood.

[10] 2. Related to this, the way in which the population dynamics are implemented in the stochastic simulations needs to be specified. Currently no information about this is provided, except for the link to the code (line 146). This is not sufficient. A more comprehensive description of the stochastic simulations is particularly important for those scenarios in the supplement that are only explored computationally (e.g., Section F). {R2:2}

We thank the referee for pointing out this lack of detail. We agree that we should have specified more precisely the way we numerically simulate the stochastic trajectories. We use the exact Gillespie algorithm to numerically simulate the population sizes. We have added a brief description of this algorithm in the methods section (lines 203ff.), and a more detailed description in the newly written Section K in the Supplementary Information.

[11] 3. What is the motivation for the choice of carrying capacity/population size (K=1000)? To me, a bacterial population of 1000 cells would seem to be rather small. Nevertheless, even for this choice the difference between stochastic and deterministic treatments is not very pronounced in most cases. Does this mean that for larger (and possibly more realistic) population sizes the deterministic theory would often be sufficient? {R2:3}

The population size of $1,000$ was chosen for computational feasibility. It does not affect the survival probability as can be seen by the (newly added) Eq. (3) in the main text, and the formulae in the Supplementary Information (Eqs. (B.8), (B.12) and (B.34)). The reason is that for the survival of resistant cells, the only deciding factor is the small resistant population size. This means that as long as the number of resistant cells at the onset of treatment is small, stochastic effects are relevant, even in (possibly infinitely) large populations.

Accordingly, the survival probability of the resistant strain is affected by the initial *density* (number divided by K), not the absolute number, of the sensitive subpopulation. This density defines the competition experienced by the initially rare resistant type. In our default parameter setting, we initialized the sensitive population size at its carrying capacity, i.e., a maximal competitive effect. If we were to decrease the initial population density of sensitive cells, the survival probability of the resistant strain would increase (intuitively, this relaxes the effect of competition on the resistant subpopulation).

To sum up, resistance evolution is a stochastic process that is not affected by the sensitive population size being large or low because the resistant individuals start at low numbers. We explain this now in the main text (line 237).

[12] 4. Lines 171 ff: The authors define "emergence" of resistance as the survival of the resistant strain until the end of the treatment and emphasize that this is not identical to the notion of "establishment" used, e.g., in Ref.22. While this is true, I wonder if in practice the two aren't essentially equivalent, in the sense that survival without prior establishment would seem to be very unlikely (it would require the resistant subpopulation to remain at small population numbers through the treatment phase). Perhaps this question could be addressed by simulations? {R2:4}

This is a very good point and exactly the reason why we also studied the size of the resistant subpopulation at the end of treatment (Fig. 4 in the main text). As we can see from that figure

(and also from one trajectory in Fig. 1), for most values of the antibiotic concentration indeed establishment and emergence coincide. However, regions of antibiotic concentrations exist where the resistant subpopulation size remains at low density. For example, in our parameterization, this occurs for concentrations slightly below $c = 10^{-2}$ (Fig. 4 – all panels). To clarify that there is a difference between establishment and emergence, we have added a reference to the section where we study the size of the resistant subpopulation when we introduce these terms (line 250).

[13] 5. Throughout the manuscript and the supplement, the MIC of the susceptible strain is shown in the concentration plots by a vertical dotted line. Although a corresponding label can usually be found in the first panel of each figure, I missed this information when first reading the manuscript. Please mention this explicitly either in the text or in the caption of the first figure where it is used. {R2:5}

We now mention the meaning of the vertical dashed line in the caption of Fig. 3 in the main text.

Reviewer 3

[14] Summary of the work: {R3:general}

In this work, the authors develop a stochastic model of a population subjected to drug treatment. This model aims to investigate the evolution of antibiotic resistance within a host. To this end, the authors focus on three quantities:

- The resistant survival probability
- The resistant sub-population size at the end of a treatment
- The carriage time after treatment

These quantities are explored by a combination of numerical and analytical tools. One of the original features of this work is the distinction between biostatic antibiotics, which impede bacterial division, and biocidal antibiotics, which kill bacteria. Another originality is that the authors distinguish two types of density regulation, either on the birth rate or on the death rate. These distinctions allowed the authors to investigate the dynamics of resistance evolution under different scenarios (e.g., biostatic antibiotic and density regulation on the death rate) and assess the impact of different antibiotic modes of action and density regulation on the evolution of resistance.

General comment:

I find this work very interesting, and I enjoyed reading it. First, given the threat to public health posed by the evolution of antibiotic resistance, this theoretical work is timely. Second, given that the development of new antibiotics is struggling, I find evaluating the impact of different drug modes of action (i.e., biostatic versus biocidal) is relevant. This aspect has been neglected, although it represents a possible angle of attack to fight antibiotic resistance. I do not have any serious concerns about this work, although I think the discussion could be strengthened (I elaborate below). I add some minor points and suggestions that the authors can address if they find them relevant.

We thank the reviewer for the positive comments and assessment of our work. We agree that a strength of our work is to highlight the effect of the antibiotic mode of action and the type of density regulation on the evolution of antibiotic resistance.

Major points:

[15] A) In my opinion, the discussion could be strengthened. If I am correct, the authors do not place their results in a clinical context. Whereas the authors claim to investigate the within-host dynamics of antibiotic resistance, they only mention in vitro experiments (lines 425-449). I think it would be great to cite some meta-analyses in which the efficiency of biostatic and biocidal drugs in eradicating a given infection is compared (e.g., Johannes Nemeth, Gabriela Oesch, Stefan P. Kuster, Bacteriostatic versus bactericidal antibiotics for patients with serious bacterial infections: systematic review and meta-analysis, Journal of Antimicrobial Chemotherapy, {R3:1}

Volume 70, Issue 2, February 2015, Pages 382–395, https://doi.org/10.1093/jac/dku379). These meta-analyses, which focus on the ability to eradicate infection and not on the evolution of resistance, do not find significant differences between biostatic and biocidal antibiotics (except in particular cases). Your work shows that there is a difference in the evolution of resistance, and it would be interesting to highlight it. In short, I think many readers will ask: is it better to use biocidal or biostatic antibiotics to prevent the evolution of resistance?

We thank the referee for pointing us to that reference. As the referee writes, we find differences in the probability of emergence of resistance between biostatic and biocidal drugs. The reference mentioned (and indeed many clinical meta-analyses) investigates the effect of drug mode of action on the outcome of infections (treatment success, patient mortality, etc.), irrespective of resistance evolution. While one could expect that biocidal drugs are more effective because they clear bacteria faster, these studies generally do not find a difference between the drug modes of action.

If the suppression of resistance evolution is a secondary goal, besides infection clearance and patient outcome, then our results suggest that biostatic drugs might be preferred over biocidal drugs. Following the suggestion of the referee, we have added a brief discussion along these lines (lines 504ff.).

[16] B) The authors compare two drug modes of action, namely biostatic and biocidal. This comparison is not novel since it has already been investigated in reference [39]. It would be interesting to compare the results of this work with those of reference [39]. Are they complementary? Do they contradict each other? Do they favor biostatic or biocidal antibiotics to prevent antibiotic resistance? Etc. I would also like to point the authors to the following reference, which is also relevant for a potential comparison: The risk of drug resistance during long-acting antimicrobial therapy, A Nande, AL Hill, Proceedings of the Royal Society B, 2022. {R3:2}

We agree with the referee that we should have compared our results more explicitly to the findings from reference [39] (now [27]), which also compares biocidal and biostatic drugs in the context of periodic antimicrobial treatment. We have now added a brief comparison to the main findings from the mentioned reference (lines 504ff.). In short, our results corroborate and extend the ones from ref. [39] to the scenario of standing genetic variation when one resistant cell is present at the beginning of treatment.

However, we refrained from discussing the paper by Nande & Hill in this context (we cite it in the introduction – see also comment [20] below) because it discusses slow and fast acting drugs (measured by absorption in a pharmacokinetic sense) in periodic treatments, a topic that does not fit well into our discussion.

[17] C) From line 395 to line 406, the authors discuss their parametrization choice. However, the authors should mention whether the values are coherent with experimental observations. For example, is a resistance cost equal to 10% relevant? The authors could cite the following meta-analysis: Melnyk AH, Wong A, Kassen R. The fitness costs of antibiotic resistance mutations. Evol Appl. 2015. Same comment for the MIC differences between resistant and sensitive bacteria. Also, I think the authors should discuss whether the resistance cost depends on the drug mode of action, as some readers may wonder if comparing biostatic and biocidal antibiotics for the same parameter values is relevant. Suppose I carry out two {R3:3}

experiments to eradicate one bacterial population: one with a biostatic antibiotic and another with a biocidal antibiotic. The evolutionary pathways to resistance will likely be different in the two cases. Here is an interesting reference: Chevereau G, Dravecká M, Batur T, Guvenek A, Ayhan DH, et al. (2015) Quantifying the Determinants of Evolutionary Dynamics Leading to Drug Resistance. PLOS Biology 13(11): e1002299.

There are three points to discuss in response to this comment by the referee: the choice of fitness cost, MIC values and evolutionary pathways depending on the drug's mode of action.

(i) *Choice of fitness costs:* We are very grateful for the reference on fitness differences between sensitive and resistant strains. In response to this comment and comment [6] by referee 1, we elaborated on our choice of parameters (new section on Parameterization – lines 159ff.). Specifically, our parameterization falls within the wide range of possible fitness effects when translated to the fitness measure of the study by Melnyk et al. (2015), who use the exponential growth difference in a specific time frame, most often 24 hours. To measure fitness differences, we have chosen to set this time to 6 hours, which corresponds approximately to the duration of the exponential phase in these competitive assay experiments. For example, if diluting a population 1/1000 fold, a division time of 30 minutes would result in an exponential growth phase of ∼5 hours ($4^t = 1000$, where t is the number of hours). Translating to our parameterization, we obtain a 6% fitness cost in our main parameterization in the main text ($\exp(\beta_R \times 0.25)/\exp(\beta_S \times 0.25)$). We added this reference in the paragraph about the parameterization (lines 159ff.) and in the discussion (line 524). Since the resistance cost, according to Melnyk et al. (2015), does not seem to differ between biostatic and biocidal drugs, we do not comment on this.

(ii) *Choice of MIC values:* We refrained from extensively commenting on MIC differences because resistance can evolve across multiple orders of magnitude (e.g. up to 1000-fold, in steps of 10-fold increases, as visualized nicely in the megaplate experiment by the Kishoni lab – `https://www.youtube.com/watch?v=plVk4NVIUh8`). We therefore believe that any choice of MIC would be justifiable, though of course smaller MIC differences are more likely to arise *de novo*. We added a short comment in line 167.

(iii) *Evolutionary pathways depending on the drug mode of action:* Lastly, we also thank the referee for the reference Chevereau et al. (2015), which we now use to motivate that the shape of the antibiotic response curves for the sensitive and resistant type are the same (line 167). However, we do not comment on potential differences of evolutionary pathways for the different drug types because Chevereau et al. (2015) do not observe a clear pattern in this respect, i.e., the level of resistance is comparable for almost all studied antibiotics. Instead, the distribution of fitness effects is largely determined by the antibiotic response curve, which we do not vary in our study.

[18] Minor points: {R3:4}

1) The authors present their model as a bacterial population within a host. I wonder whether it is necessary to specify "within a host." My concern is that it is unclear what distinguishes the authors' model from a model of an in vitro bacterial population. Also, as specified from line 419 to line 424, the authors' model does not include pharmacokinetics, an essential ingredient of within-host bacterial populations subjected to antibiotics.

We agree with the reviewer that our model is not specific for real hosts, i.e., *in vivo* dynamics. By writing *within-host*, we wanted to highlight the difference to models of antibiotic resistance evolution at the host population level, i.e., the transmission of resistant and sensitive strains *between hosts*. Based on this comment, and comment [6] from Reviewer 1, it is clear that our use of the terminology *within host* has been misleading. We have therefore changed the title, also based on the next comment [19] by this reviewer. Additionally, we have been more careful with the use of the term *within host* throughout the manuscript and avoided using it when possible.

[19] 2) Instead of specifying the quantities the authors focus on in the title, why do they not mention the comparisons they made, namely different drug modes of action and types of density regulation? This would make the title more attractive. {R3:5}

In response to this comment, and to comment [6] by Reviewer 1, we have changed the title to "A stochastic analysis of the interplay between antibiotic dose, mode of action, and bacterial competition in the evolution of antibiotic resistance". We agree with this reviewer that the focus on within-host models was misleading in this context (see also our response to comment [18]). We meant to separate our model from between-host models that study antibiotic resistance on the population level. We also agree that a large part of our work addresses the differences between the two drug types, which is now better reflected in the title. This is now better represented in the title.

[20] 3) lines 32-33: I suggest the authors go through the following paper: The risk of drug resistance during long-acting antimicrobial therapy, A Nande, AL Hill, Proceedings of the Royal Society B, 2022. {R3:6}

We thank the author again for this reference. We have now specified that (in at least) two papers (Marrec and Bitbol, 2020; Nande and Hill, 2022), numerical solutions of the resistant survival probability have been obtained (line 49). However, we would like to stress that so far no explicit form of the survival probability like in Eq. (3) (we now added this equation to the main text) had been derived.

[21] 4) lines 63-64: Maybe I am wrong, but this sentence sounds like the resistant sub-population goes extinct with a probability equal to one in the drug-free phase. The extinction probability is not equal to one, right? If the resistant sub-population size is substantially larger than the sensitive sub- population size at the end of treatment, the latter would probably die out even if the sensitive individuals have a selective advantage, right? Could the authors provide the reader with an idea of the extinction probability? {R3:7}

The reviewer is correct. In our stochastic model, extinction of the resistant type is not guaranteed in all generality. However, we assumed that resistant cells get extinct after treatment. Indeed, our analysis of extinction time is most relevant for commensal bacteria, which after a brief bout of antibiotic treatment do not experience treatment for an extended time period. In such scenarios, the constant transmission (influx) of new bacterial cells into the gut results in the eventual invasion of one sensitive cell.

We chose to summarize this process by conditioning the resistant dynamics on extinction after treatment. The transition rates are theoretically transformed to describe trajectories that

will eventually result in the loss of the resistant strain (details are provided in the Supplementary Information, Section E, e.g. Eq. (E.4)). We note however, that we did not change the simulations, but accounted for this conditioning by only recording trajectories where the resistant type was replaced by the sensitive strain. We have added a very brief clarification about repeated re-introduction events in line 80: "The sensitive strain eventually, potentially after repeated re-introductions, competitively excludes the resistant subpopulation because of the cost of resistance."

[22] 5) line 91: "the overall birth and death rate" = the per capita birth and death rate? {R3:8}

We have followed this suggestion and changed the wording accordingly (line 113).

[23] 6) line 116: I would replace "maximal growth rate" with "net growth rate." In microbiology, the maximal growth rate usually refers to the maximum of the curve (dN/dt)/N, which is, in the case of a lag-free logistic growth, obtained at time t=0 and not "measured during the exponential phase" (line 116). {R3:9}

We have followed this suggestion (line 138).

[24] 7) Figure 2: The authors could add "grow" above 0 and "decline" below 0 to help the reader identify the antibiotic concentration regimes leading the bacterial population to extinction. {R3:10}

We have added these labels to Fig. 2.

[25] 8) line 136: The word "cell" is lost all by itself in a line. {R3:11}

We have corrected this typo.

[26] 9) lines 137-146; Stochastic simulations: More information could be brought into this section. For example, what algorithm did the authors make use of? Is it a Gillespie algorithm, which is exact, or did the authors use an approximate method such as tau-leaping? Also, it would be useful to provide a pseudo-code where you specify every step of your algorithm (i.e., initialization, iteration, etc.) for readers unfamiliar with C++. The advantage of writing a pseudo-code is to allow every reader to write their code with their favorite programming language. The pseudo-code does not need to be in the main text but could be in the supporting information. {R3:12}

We have added a short description of our simulation procedure (exact Gillespie algorithm) in the main text (line 203; see also our response to comment [10] by Reviewer 2). Additionally, we have outlined the details about the exact Gillespie simulation in two specific scenarios in the Supplementary Information (new Section K) that should help readers to reproduce the simulations in any programming language.

[27] 10) lines 162-163: I was very surprised by this sentence. How can standing genetic variation and de novo mutations yield similar results? This sentence may sound weird to any reader with the following reasoning: a perfect biostatic antibiotic fully prevents sensitive microbes from dividing so that the sensitive birth rate equals 0. In such a case, it is clear that {R3:13}

no de novo mutants appear. A biocidal drug does not prevent sensitive microbes from dividing. Although it drives the sensitive bacterial population to extinction faster than a perfect biostatic drug (at least for large enough drug concentrations), a large mutation rate could induce some appearances of mutants, which would find themselves in a situation in which they are favored by natural selection since they are less impacted by the drug than the sensitive microbes. Could the authors comment on that?

We are very grateful for this comment. It made us realize that we had plotted wrong simulation results in most of the panels in Figs. F.1-6 in the Supplementary Information. We have corrected this now. We now show results that agree with the intuition of the referee. Precisely, biostatic drugs at high concentrations prevent cell replication and by that prevent any mutations to appear, which is visible now in the figures (blue symbols are exactly at zero for antibiotic concentrations above the sensitive MIC). We comment on these points starting in line 229.

[28] 11) lines 165-167: The authors could add that a deterministic approach does not allow extinction modeling. {R3:14}

We have added this specification (line 241).

[29] 12) Figure 3: Could the authors add to the caption over how many stochastic realizations the data points are averaged? I know the authors wrote in line 143 that the number of stochastic realizations equals 10^6, but including it in a caption is important. Also, could the authors confirm that $\gamma = \gamma_S = \gamma_R$ and $X_R(0) = 1$? Are there too many parameters to be added to the caption instead of "Parameter values are the same as in Fig. 2"? Same comment for the other figures. {R3:15}

We have added the suggested details to the legend of Fig. 3 and the number of stochastic simulations to each figure legend. However, we refrained from repeating all parameter values in all the figure legends, which in our opinion is unnecessary duplication of information. All default parameter values can now be found in the caption of Fig. 3 and additionally in Table A.2 in the Supplementary Information.

[30] 13) lines 365-366: Could the authors remind the reader of how they scale? Also, the word "intrinsic growth rate" is unclear to me. Is it the difference between the intrinsic birth and death rates? {R3:16}

We agree with the reviewer that our phrasing was not clear. In response to this comment, we have rewritten this sentence as follows (line 470): "The distance between the sensitive MIC and the maximizing concentration is determined by the death rate of the sensitive strain and the shape of the antibiotic response curve of the sensitive strain.". In particular, we do not use the words *scale* and *intrinsic* anymore.

[31] 14) lines 385-388: This sentence is quite long, and I am not sure I understood it. It is not clear to me how it supports the previous sentence. There is a similar reasoning in lines 186-188. How can two populations have the same deterministic dynamics if one has large birth and death rates and the other has low birth and death rates? {R3:17}

In response to this comment we have split the long sentence into two shorter sentences and added some clarification about the connection to the previous sentence (line 494). The reason for this observation is that the same deterministic dynamics can have different stochastic fluctuations, which is explained by the scaling of the stochastic fluctuations, e.g. Parsons et al. (2018) (and more generally the theory of stochastic differential equations or the master equation in physics). For example, consider a hypothetical strain 1 with birth rate $\beta_1 = 4$ and death rate $\delta_1 = 3.8$, and a hypothetical strain 2 with birth rate $\beta_2 = 0.4$ and death rate $\delta_2 = 0.2$. Then the deterministic dynamics $(\beta_i - \delta_i)$ of these two strains are the same. However, the stochastic fluctuations, which scale with $\beta_i + \delta_i$, are much larger for strain 1 than for strain 2. This is why strain 1 has a larger extinction risk than strain 2.

[32] 15) lines 390-392: This sentence made me realize that the authors do not provide insight into the mean extinction time of a sensitive bacterial sub-population subjected to antibiotics. Since the authors claim that biocidal antibiotics drive sensitive sub-populations to extinction faster than biostatic antibiotics, it would be interesting to provide some timescales. For example, the mean extinction time under a perfect biostatic antibiotic is given approximately by $\log(K)/\delta_S$. How does the mean extinction time under biocidal antibiotics compare to $\log(K)/\delta_S$? Do biocidal antibiotics drive the sensitive sub-population to extinction faster than biostatic antibiotics at all drug concentrations? {R3:18}

In Fig. A.1 in the Supplementary Information, we show that biocidal antibiotics can drive sensitive bacterial populations to extinction faster than biostatic antibiotics, especially for high antibiotic concentrations. The reason is that as soon as the antibiotic effect is larger than the sensitive birth rate, $\alpha_S(c) > \beta_S$, a biostatic drug has reached its maximal effect, which is not the case for a biocidal drug, where the death rate can increase further. The exponential decline rate is therefore given by $-\beta_S + \delta_S + \alpha_S(c)$ in the biocidal case and by $\delta_S - \max(0, \beta_S - \alpha_S(c))$ in the biostatic case (which reduces to δ_S if $\alpha_S(c) > \beta_S$). Therefore, the two drug treatments start to differ deterministically if $\alpha_S(c) > \beta_S$, in the absence of potential effects of density regulation. In this case, biocidal drugs eradicate the sensitive bacterial population faster than biostatic drugs.

We have added a reference to Fig. 2 and to Fig. A.1 at the end of the sentence that the reviewer mentions (line 501). However, we do not go into details of the time scales because this would be too technical in the main text. Instead, we added this time-scale discussion in the Supplementary Information (around Fig. A.1).

References

Alexander, H. K. and MacLean, R. C. Stochastic bacterial population dynamics restrict the establishment of antibiotic resistance from single cells. *Proceedings of the National Academy of Sciences*, 117(32):19455–19464, 2020. doi: 10.1073/pnas.1919672117.

Ankomah, P. and Levin, B. R. Exploring the collaboration between antibiotics and the immune response in the treatment of acute, self-limiting infections. *Proceedings of the National Academy of Sciences*, 111(23):8331–8338, 2014. doi: 10.1073/pnas.1400352111.

Brown, S. P., Inglis, R. F., and Taddei, F. SYNTHESIS: Evolutionary ecology of microbial wars: within-host competition and (incidental) virulence. *Evolutionary Applications*, 2(1):32–39, 2009. doi: 10.1111/j.1752-4571.2008.00059.x.

Chevereau, G., Dravecká, M., Batur, T., Guvenek, A., Ayhan, D. H., Toprak, E., and Bollenbach, T. Quantifying the determinants of evolutionary dynamics leading to drug resistance. *PLoS Biology*, 13(11):e1002299, 2015. doi: 10.1371/journal.pbio.1002299.

Day, T. and Read, A. F. Does high-dose antimicrobial chemotherapy prevent the evolution of resistance? *PLoS Computational Biology*, 12(1):e1004689, 2016. doi: 10.1371/journal.pcbi.1004689.

Granato, E. T., Meiller-Legrand, T. A., and Foster, K. R. The evolution and ecology of bacterial warfare. *Current biology*, 29(11):R521–R537, 2019.

Grant, A. J., Restif, O., McKinley, T. J., Sheppard, M., Maskell, D. J., and Mastroeni, P. Modelling within-host spatiotemporal dynamics of invasive bacterial disease. *PLoS Biology*, 6(4):e74, 2008.

Hibbing, M. E., Fuqua, C., Parsek, M. R., and Peterson, S. B. Bacterial competition: surviving and thriving in the microbial jungle. *Nature reviews microbiology*, 8(1):15–25, 2010.

Marrec, L. and Bitbol, A.-F. Resist or perish: Fate of a microbial population subjected to a periodic presence of antimicrobial. *PLoS Computational Biology*, 16(4):1–19, 2020. doi: 10.1371/journal.pcbi.1007798.

Melnyk, A. H., Wong, A., and Kassen, R. The fitness costs of antibiotic resistance mutations. *Evolutionary applications*, 8(3):273–283, 2015.

Monod, J. The growth of bacterial cultures. *Annual review of microbiology*, 3(1):371–394, 1949.

Nande, A. and Hill, A. L. The risk of drug resistance during long-acting antimicrobial therapy. *Proceedings of the Royal Society B: Biological Sciences*, 289(1986), 2022. doi: 10.1098/rspb.2022.1444.

Niehus, R., Oliveira, N. M., Li, A., Fletcher, A. G., and Foster, K. R. The evolution of strategy in bacterial warfare via the regulation of bacteriocins and antibiotics. *eLife*, 10, 2021. doi: 10.7554/elife.69756.

Parsons, T. L., Lambert, A., Day, T., and Gandon, S. Pathogen evolution in finite populations: slow and steady spreads the best. *Journal of The Royal Society Interface*, 15(147):20180135, 2018. doi: 10.1098/rsif.2018.0135.

Poulsen, L. K., Licht, T. R., Rang, C., Krogfelt, K. A., and Molin, S. Physiological state of Escherichia coli BJ4 growing in the large intestines of streptomycin-treated mice. *Journal of Bacteriology*, 177(20):5840–5845, 1995.

Regoes, R. R., Wiuff, C., Zappala, R. M., Garner, K. N., Baquero, F., and Levin, B. R. Pharmacodynamic functions: a multiparameter approach to the design of antibiotic treatment regimens. *Antimicrobial Agents and Chemotherapy*, 48(10):3670–3676, 2004. doi: 10.1128/aac.48.10.3670-3676.2004.

Scire, J., Hozé, N., and Uecker, H. Aggressive or moderate drug therapy for infectious diseases? Trade-offs between different treatment goals at the individual and population levels. *PLoS Computational Biology*, 15(8):e1007223, 2019. doi: 10.1371/journal.pcbi.1007223.

Tedijanto, C., Olesen, S. W., Grad, Y. H., and Lipsitch, M. Estimating the proportion of bystander selection for antibiotic resistance among potentially pathogenic bacterial flora. *Proceedings of the National Academy of Sciences*, 115(51), 2018. doi: 10.1073/pnas.1810840115.
